# Frontal Sinus Morphological and Dimensional Variation as Seen on Computed Tomography Scans

**DOI:** 10.3390/biology11081145

**Published:** 2022-07-29

**Authors:** Austin A. Shamlou, Sean D. Tallman

**Affiliations:** 1 Department of Anatomy & Neurobiology, Boston University School of Medicine, 72 E. Concord St. L1004, Boston, MA 02118, USA; ashamlou@bu.edu; 2 Department of Anthropology, Boston University, Boston, MA 02215, USA

**Keywords:** forensic anthropology, elliptical Fourier analysis, computed tomography scans, human variation, climactic adaptation, sexual dimorphism

## Abstract

**Simple Summary:**

The frontal sinus is an important cavity inside an individual’s forehead and has been used by forensic anthropologists to provide positive identifications due to its highly unique structure from person to person, yet researchers still do not fully understand why it forms as it does. This study examined the differences in both shape and size of the frontal sinuses of over 300 individuals from various ancestral backgrounds and assigned sexes to see if climate adaptations or sexual dimorphism might be driving factors. Results showed that shape was not dependent on where a person descended from nor their assigned sex at birth; however, dimensionally, these variables in combination do cause some significant variation. The results also speak to the idiosyncratic nature of the frontal sinus and bolster confidence in using morphological variations as a means of personal identification. While it is still unknown what causes the significant shape variation between individuals within the U.S., it appears that the frontal sinus is affected more by sexual dimorphism than by the ancestry of the individual.

**Abstract:**

Frontal sinus variation has been used in forensic anthropology to aid in positive identification since the 1920s. As imaging technology has evolved, so has the quality and quantity of data that practitioners can collect. This study examined frontal sinus morphological and dimensional variation on computed tomography (CT) scans in 325 individuals for assigned sex females and males from African-, Asian-, European-, and Latin American-derived groups. Full coronal sinus outlines from medically derived CT images were transferred into SHAPE v1.3 for elliptical Fourier analysis (EFA). The dimensional data were measured directly from the images using the MicroDicom viewer. Statistical analyses—Pearson’s chi-square, ANOVA, and Tukey post hoc tests—were run in R Studio. Results indicated that 3.7% lacked a frontal sinus and 12.0% had a unilateral sinus, usually on the left (74.3%). Additionally, no statistically significant morphological clustering using EFA was found based on assigned sex and/or population affinity. However, there were statistically significant differences dimensionally (height and depth) when tested against assigned sex and population affinity, indicating that the interactive effects of sexual dimorphism and adaptive population histories influence the dimensions but not the shape of the frontal sinus.

## 1. Introduction

Positive identification via antemortem-postmortem radiographic comparison has been an important aspect in forensic anthropology research and casework [1,2,3,4,5,6,7,8,9,10,11,12,13,14,15,16,17,18,19,20,21,22]. In particular, the frontal sinus has been found to be unique to an individual and likened to fingerprints in terms of individuation; even monozygotic twins do not present identical frontal sinus morphologies [23]. Radiographic comparison of frontal sinuses has been used for positive identification for almost a century, often used when dental or other medical records are missing, if the mandible and maxilla are not recovered, and in especially challenging identifications [3,4,9,12,13,24,25,26]. Despite its importance in establishing positive identifications, little is known about the factors that contribute to frontal sinus morphological variation and uniqueness, which presents challenges to expert witness testimony [2]. Thus, this study explores whether sexual dimorphism and/or population affinity impact the shape and size of frontal sinuses.

### 1.1. Morphology, Development, and Function of the Frontal Sinus

The frontal sinus presents typically as two asymmetrical cavities in the frontal bone [13,19,27,28,29,30,31]. These air-filled and mucous-lined cavities connect to the nasal cavity and extend superiorly from the anterior ethmoid sinus [32]. It is not uncommon for individuals to exhibit bilateral asymmetry in sinus formation or lack a cavity on either the left or right side—around 5% of the population lacks a frontal sinus entirely [13,19,27,28,29,30,31]. The periphery of the frontal sinus can present in a scalloped pattern with unpredictable outlines, and the size and depth of the frontal sinus are also highly variable.

The frontal sinus begins formation during the fourth or fifth fetal month and is actively developing at two or three years of age [3,27]. By the fourth or fifth year of life, the frontal sinus can be observed on radiographs and it continues to develop and change morphologically throughout puberty, with left and right cavities developing independently [12,27]. Development is typically complete by 20 years and remains stable throughout adulthood, barring significant trauma, chronic illness targeting the sinuses, tumors, or reabsorption in extreme old age [23,33,34,35].

Functions of the frontal sinus are postulated to include regulation of the respiratory system, relieving pressure within the body, lightening the weight of the cranium, and/or aiding in thermal regulation [3,9,27,36,37,38]. It is hypothesized that the frontal sinus may be affected by extreme respiration, forcing the frontal sinus as well as the paranasal sinuses to adapt in volume as the body regularly increases/decreases in heat and internal pressure [36,39]. In panting animals, it is thought that the negative space within the cranium acts as a larger area for evaporation while avoiding respiratory alkalosis [40]. For humans and other non-panting species, this function occurs as a mechanism of thermal homeostasis instead of targeted brain cooling [41]. This occurs when the individual breathes rapidly and lets out an excess of carbon dioxide which leads to an increased pH level in the blood [42]. The frontal sinus and other cavities within the cranium allow for more efficient inhalation of O_2_ without being energetically taxing [40].

Researchers have postulated that sinus volume or morphology may be affected by adaptations to climate or due to sexual dimorphism [3,27,37,38]. However, these studies produced varying results, with the majority finding that in European and Neanderthal individuals (i.e., cold-adapted populations), the frontal/paranasal sinuses are hyperpneumatized (i.e., expanded) in comparison to African individuals (i.e., warm-adapted populations) [38,43]. It is possible that frontal sinuses work in conjunction with other craniofacial structures as adaptations to varied climates; however, the specific role of the frontal sinuses ultimately remains unknown [37,38]. Studies have noted statistical significance when assessing approximate dimensions and bilateral absences against population groups located in different continents, which infers that the frontal sinus develops variably in environments of varying climate conditions [27,33].

Limited research has also found the frontal sinus to be potentially moderately sexually dimorphic given that the frontal is typically more robust in males due to males having larger crania [33,35,36,44]. Although, authors have noted that the contour morphology and outer limits of the sinuses are likely due to environmental or genetic variations rather than due to sexual dimorphism [26,33]. Hacl et al. [36] proposed that the frontal sinus’s role in ventilation likely contributes to the unique features of the shape, expressing that higher ventilation and increased internal pressure of individuals would affect morphology. Hamed et al. [35] used CT scans for their assessment of the reliability of the frontal sinus in estimating sex and found that sinus depth was marginally discriminatory. They hypothesized that radiographs skewed the information by flattening 3D structures into a 2D image. They further hypothesized that radiographs would be beneficial to assess density, but CT would be better for examining the intricacies of bone morphology.

### 1.2. Visual Comparison and Superimposition

Visual comparison and superimposition methods have been the best and most frequently used methods when assessing frontal sinuses in order to positively identify remains [23,26]. Numerous case studies have demonstrated the success of these methods [4,9,12,13,45]. Anthropologists have attempted to create other methods, such as metric analyses and various coding systems based on specific parameters of the frontal sinus to aid in positive identification; however, these have only been successful in excluding individuals [13,19,28,30,34,46].

Increasingly, radiologists and anthropologists look for the detail and clarity that CT or magnetic resonance imaging (MRI) scans can provide over traditional radiographs [24]. With more detail, it is presumed that more reliable positive identifications can be made as the number of concordances exponentially increases. Additionally, 3D-CT techniques eliminate the issue of discrepancies in angularity [47]. With the whole element or region captured in overlapping slices, practitioners are able to move the CT images to align more easily than when they had to mimic compressed 2D renderings.

However, there has been a call for forensic anthropology to create more stringent standards for this methodology in order to maintain validity in court [6,29,48]. This has been accomplished, in part, through elliptical Fourier analysis (EFA), which is used to capture the intricacies of a closed shape and to translate this morphological data into quantitative data [29,49,50,51]. Previous studies on EFA have been used to assess variation in fossils, insect wings, fish, sclerites, and stone tools [52,53,54,55,56,57]. Anthropologists have primarily focused on craniofacial morphology including assessments of the mandible, orbits, nasal aperture, dentition, overall facial profile, and facial soft tissues [58,59,60,61,62,63,64,65,66,67]. Postcranially, anthropologists have used EFA to study sexual dimorphism in the proximal humerus and pelvis, pair matching, and positive identification using the clavicle, vertebrae, and frontal sinuses [2,29,68,69,70]. Previous studies of the frontal sinus and EFA have addressed overall skull morphology quantified the uniqueness of the frontal sinuses, and established error rates in matching frontal sinuses [2,3,29,34,49].

Because the roles of population affinity and sexual dimorphism in frontal sinus morphological variation are not fully understood, the current study uses morphological and dimensional analyses of medically derived CT scans from a diverse and robust sample of living individuals from the United States. It is hypothesized that frontal sinus morphological variation will show subtly patterned clusters along assigned sex and population affinities and that there will be statistically significant differences in the dimensions of the frontal sinus between assigned sex and some population affinity groups. These differences are presumably related to sexual dimorphism and differing ancestral population histories included in the study sample (i.e., individuals descended from differing biogeographic groups representing varying climatic conditions), and not attributed to the social race categories used in medical and bureaucratic administration. For example, individuals descended from colder adapted populations may be subtly different from those descended from hotter adapted populations. As such, the morphometric variation will likely overlap between bureaucratic groups and is not intended to be used in predictive assigned sex or social race group classifications.

## 2. Materials and Methods

We must note the terminology used in this study as there is significant variation in the literature. In order to discuss possible climate adaptations within the frontal sinus, proxies for geographic variation were used on the sample. While imprecise, this proxy is described using the term “population affinity”—referred to as “ancestry” in many studies—and samples herein are designated based on where an individual is biogeographically derived from: African-derived, Asian-derived, European-derived, and Latin American-derived. Terms like “race” have been used in previous studies; however, race is a social concept and does not adequately account for or explain human biological variation [71]. Historically, forensic anthropologists have used “ancestry” synonymously with social race and have used terms like “Hispanic” to describe an individual’s ancestral lineage. Now, these terms and classifications of human groups are being critiqued and reanalyzed, and there has been a shift in practitioners’ perspectives regarding the efficacy and role of ancestry estimation [71,72,73,74]. Similarly, the discussion of assigned sex, gender, and the terminology surrounding these categories is currently being evaluated [75,76]. Most of the recent literature discusses skeletal or osteological sex as “biological sex” in regard to any sex estimations made by forensic anthropologists. However, this study will use “assigned sex”, “assigned female at birth (AFAB)”, and “assigned male at birth (AMAB)” as they are the most trans-inclusive and signify that sex is assigned at birth and can change.

The current study assesses frontal sinus morphological variation using EFA of the outermost limits of the frontal sinus as seen on CT scans. Additionally, this study examines the variation of frontal sinus dimensions (e.g., maximum height, maximum width, and maximum depth, and the product of those variables). All CT scans in this study were provided by the Boston Medical Center’s (BMC) Radiology Department, and this study was deemed exempt through Boston University’s Institutional Review Board (#H-39711). Anonymized images were provided via the HIPPA-compliant cloud management service, “Box”, and protected health information was stored on a HIPPA-compliant BMC-controlled computer. The first author was provided a master code spreadsheet that included anonymized participants, their assigned sexes, and their population affinities. No identifiable information or medical history was provided to the authors. Clinically standardized coronal and sagittal plane CT image slices with 1.5 mm thicknesses were obtained from living adults aged 20–50 years who had no history of trauma to the frontal area and no history of chronic illness within the frontal sinus. Individuals with clearly rotated, raised, or lowered skulls were removed from analysis to minimize the distorting effects of deviated skull orientation. While all individuals received cranial imaging for various health-related assessments, they did not receive medical care for frontal sinus-related issues. Scans were initially obtained from 325 AFABs and AMABs from African-, Asian-, European-, and Latin American-derived population affinities (Table 1). The study sample does not represent the entirety of frontal sinus morphological variation; however, such groups are commonly identified in the clinical record and may reflect ancestral adaptations to a variety of temperate, subtropic, and tropic environments from which they are descended. Prior to morphological or dimensional analyses, individual CT scans were assessed to identify those that lacked a frontal sinus and those who presented with unilateral sinuses. Individuals with frontal sinuses that did not connect completely medially were included in the dimensional analysis but not the morphological analysis (Table 1). If the frontal sinus was unilateral, the presenting side was noted.

### 2.1. Morphological Analysis

**Figure 1:**
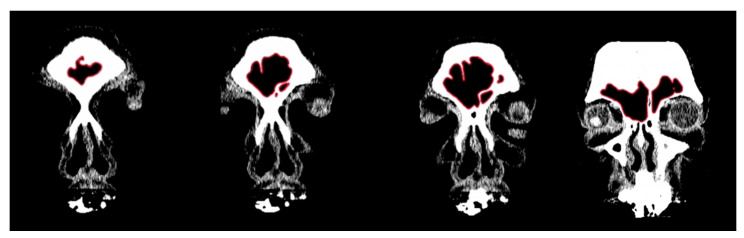
Example of CT image slices from a study individual with the frontal sinuses traced in red in Adobe Photoshop.

**Figure 2:**
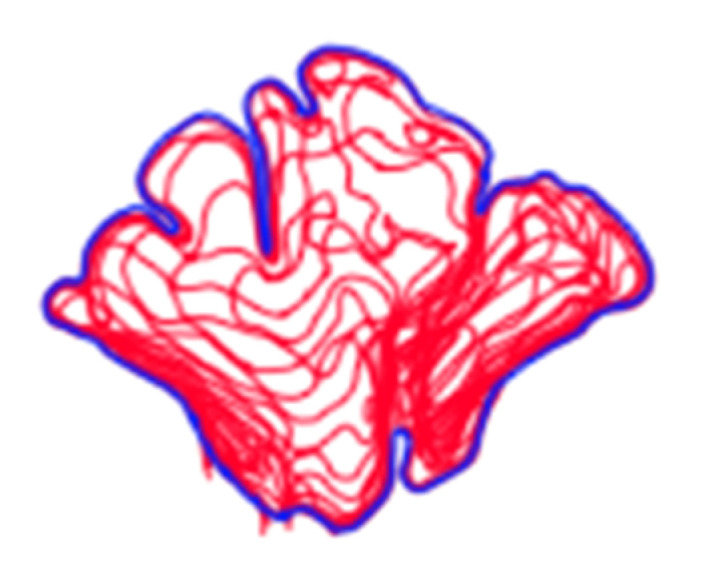
Example of a composite assemblage of all frontal sinus slices (*n* = 15) traced in red with outermost limit in blue. Note that the red lines extending from the inferior border represent the sinus’s transition into the nasal cavity.

**Figure 3:**
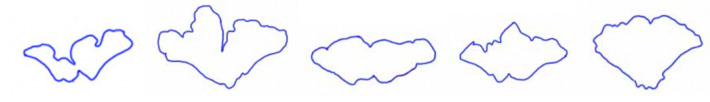
Examples of outermost blue outlines of frontal sinuses saved as BITMAP files.

In order to conduct a standardized morphological analysis of frontal sinus variation on CT scans, the outermost shape detail was captured by compressing the 3D images of the CT scan slices into one overlapping 2D shape. Using the MicroDicom viewer, image slices were identified that displayed portions of the frontal sinus in a clinically standardized anteroposterior view on the coronal plane. Between 15 and 20 image slices were obtained for each frontal sinus due to dimensional variation in order to visualize the structure. Additionally, as a buffer, the image slice immediately preceding (anterior) and following (posterior) the frontal sinus were also downloaded. This group of image slices was assembled in Adobe Photoshop with each slice occupying a separate layer and accompanied by a transparent layer for image tracing. For visibility, 70% red opacity was used with a 2-pixel wide brush to trace each layer. The traced image for each layer included the outermost borders of the frontal sinus that can be seen on each slice (Figure 1 and Video S1). Once all of the CT slices were traced, the CT images were hidden, and all of the transparent layers were made visible (Videos S1 and S2). This created a compressed image of the layered outermost limits of the frontal sinus. Next, a final transparent layer was created on top of all traced layers using 100% blue opacity with a 2-pixel width brush to create a final outline of the outermost limits of this layered shape (Figure 2 and Video S2). Unlike radiographic imaging, the inferior border of the frontal sinus was clearly delineated in the CT slices. If there were a few incomplete red lines below the inferior blue border, these represent areas where the frontal sinus transitions into the nasal cavity or ethmoid sinus and were not considered part of the overall outline of the frontal sinus (see Figure 2). Finally, all layers were made invisible except the final blue outline, which was saved as a BITMAP (.bmp) file that could be read in the SHAPE v1.3 software (Figure 3). In order to conduct an EFA in SHAPE v1.3, the outlines of the frontal sinuses must be a complete, single-enclosed shape; thus, the sample size was reduced from 325 to 307 (see Table 1) [77]. 

**Figure 4:**
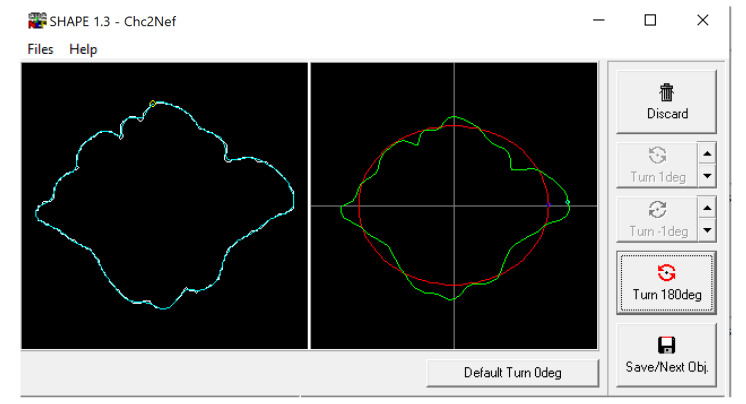
Example of frontal sinus outline (**left**) and accompanying analysis in the SHAPE v1.3 software using Chc2Nef (**right**). The software calculates coefficients to alter an ellipsis (red) to approximate the frontal sinus outline (green).

The BITMAP files were processed through the ChainCoder, Chc2nef, and PrintComp programs [78] (Figure 4). This software performs an EFA as it interprets the closed shapes and provides coefficients that can be used as quantitative data to assess patterns amongst groups [2]. This study associated these coefficients with morphological cluster groups and compares them against assigned sexes and population affinities using Pearson’s chi-square and analysis of variance (ANOVA) tests in R Studio.

### 2.2. Dimensional Analysis

**Figure 5:**
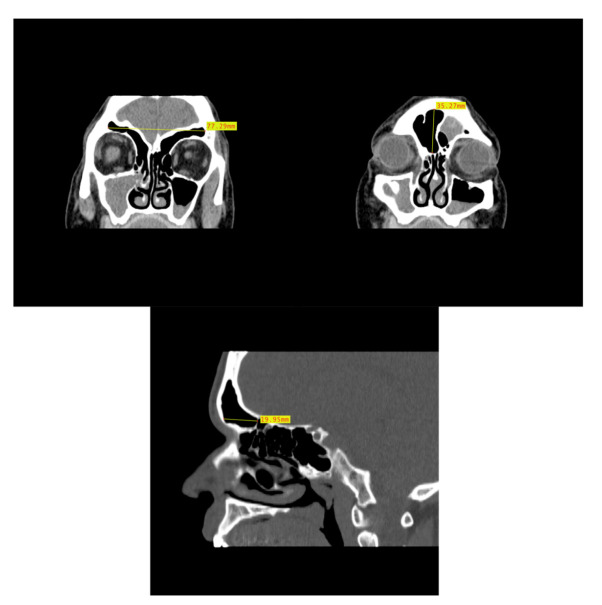
Example of CT image slice showing the maximum height (**left**) and width (**right**) measurement of the frontal sinus in the coronal plane and the frontal sinus maximum depth (**bottom**) in the sagittal plane from three study individuals (yellow lines and red boxes).

In order to assess the dimensional variation based on assigned sex and population affinity, the maximum heights, widths, and depths of each frontal sinus were examined individually and in combination. In order to capture the dimensions, the MicroDicom viewer was used because the Shape v1.3 software used in the morphological analysis lacks scaling capabilities. While viewing the images in the MicroDicom viewer, linear measurements in millimeters were obtained to the hundredths. The maximum height and maximum width of the frontal sinus were obtained from images in the coronal plane, while the maximum depth was taken from slices in the sagittal plane (Figure 5). Additionally, the product of height, width, and depth (H × W × D) was used as a rough proxy for volume; however, we acknowledge that this dimension does not accurately represent the contoured volume of sinuses. The measurements were recorded in a Microsoft Excel spreadsheet with the individual’s study ID number, assigned sex, and population affinity.

### 2.3. Statistical Analysis

To determine the significance of the morphological variation, a subset of the raw data was transformed before statistical analyses were conducted. The output produced by SHAPE v1.3 is a Nikon Electronic Format (NEF) file with coefficients that represent the shape of each individual’s frontal sinus. In order to run a statistical analysis on these data, a principal component analysis (PCA) was run in R Studio using the “mclust” extension package. The PCA reduced the data from the independent coefficients per unique shape to three dimensions that the shapes were then categorized into. These three dimensions represented clusters of similar shapes, and each individual frontal sinus was assigned a cluster classification labeled 1–3 following this analysis. These clusters were then compared against the assigned sexes and population affinities using Pearson’s chi-square tests in R Studio. Statistically significant relationships were identified when the *p*-value was <0.05. Additionally, ANOVA tests were run to determine if assigned sex and population affinity clustered significantly. ANOVA tests with a *p*-value of the F-statistic below 0.05 were reported as statistically significant. Pearson’s chi-square and ANOVA tests were conducted on the dimensional data using the same parameters to determine statistical significance. Tukey post hoc tests were then run on the statistically significant ANOVA results.

## 3. Results

**Figure 6:**
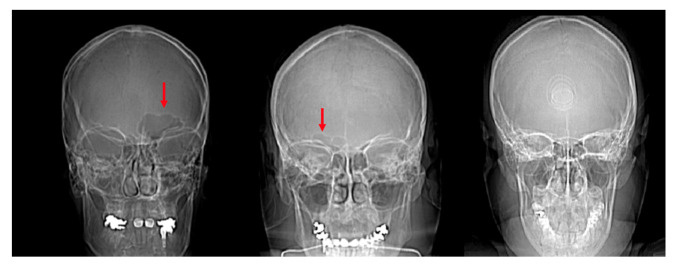
Scout images of the crania of individuals with a unilateral left frontal sinus (**left**, red arrow), unilateral right frontal sinus (**center**, red arrow), and bilateral absence of a frontal sinus (**right**).

The frontal sinus was absent in 12 individuals (3.7%) and six (1.8%) lacked a frontal sinus that intersected medially. As described above, this reduced the sample to 307 individuals for the morphological analyses (see Table 1). The individuals lacking a frontal sinus included three African-derived AFABs, two European-derived AFABs, two Latin-American derived AFABs, two Asian-derived AMABs, and two European-derived AMABs. Additionally, out of the 325 individuals assessed, 39 (12.0%) had unilateral frontal sinuses. There were 29 individuals (74.4%) that had frontal sinuses developed on their left sides and 10 individuals (25.6%) who had sinus development on the right (Figure 6). There were no significant differences between left and right presentations vs. assigned sexes or population affinities. However, a two-sample *t*-test indicated that left presentations are more common than right (*t* = 3.7025, df = 6, *p*-value = 0.01006).

### 3.1. Shape Variation Analysis

Pearson’s chi-square tests for assigned sex (X^2^ = 3.3066, df = 2, *p*-value = 0.1914) and population affinity (X^2^ = 6.247, df = 6, *p*-value = 0.3961) vs. the three-group EFA cluster classification identified from SHAPE v1.3 indicate no statistically significant clustering of frontal sinus morphology based on assigned sex or population affinity. Likewise, when running an ANOVA on assigned sex and population affinity vs. EFA cluster classification, there is no statistical significance between these factors (Residual Deviance = 164.48, df = 3, *p*-value = 0.5373).

### 3.2. Dimensional Variation Analysis

Results from Pearson’s chi-square and ANOVA tests are presented in Table 2 and Table 3. None of the Pearson’s chi-square tests were statistically significant, indicating that neither assigned sex nor population affinity alone affects the dimensional variables. However, the results of the ANOVA tests indicate that when assigned sex and population affinity are tested against the variables of maximum height, maximum depth, and H × W × D, there are statistically significant differences between at least two groups. Maximum width did not exhibit sexual dimorphism or population affinity differences. When running Tukey post hoc tests on the statistically significant ANOVA results, assigned sex and assigned sex in combination with population affinity demonstrated the highest impact, while no population differences among AFABs or among AMABs were observed (Table 4). In particular, maximum depth was the most sexually dimorphic trait, and differences were found between African-derived AFABs and AMABs and Latin American-derived AFABs and AMABs, in addition to seven other population affinity/assigned sex comparisons. The product of all three dimensions produced the next most sexually dimorphic results, with differences found between African-derived AFABs and AMABs and three other population affinity/assigned sex comparisons. Lastly, maximum height was the least sexually dimorphic trait with three statistically significant population affinity/assigned sex comparisons; however, no single population affinity demonstrated sexual dimorphism in sinus height.

Frontal sinus heights ranged from 7.02 mm in an Asian-derived AFAB to 58.81 mm in an Asian-derived AMAB. The maximum widths ranged from 9.95 mm in an Asian-derived AFAB to 114.02 mm in a European-derived AFAB. The maximum depths ranged from 3.65 mm in an Asian-derived AFAB to 28.26 mm in a Latin American-derived AMAB. Table 5, Table 6 and Table 7 include the mean, range, sample size, and standard deviation for each group. Typically, the overall range within one population affinity group was larger than the range for either AFAB or AMAB groups alone within that population affinity. However, in the African-derived population affinity group, the total population range was equal to the range of the AMAB group alone, indicating that African-derived AMABs measured both the absolute minimum and maximum while the African-derived AFABs presented more intermediate. This occurred in all three dimensions; maximum height, width, and depth (see Table 5, Table 6 and Table 7). Asian-derived AFABs had the same range as all Asian-derived individuals when measuring the maximum width, indicating Asian AFABs had the largest variation (Table 6).

## 4. Discussion

### 4.1. Frontal Sinus Absence and Unilateral Expression

This study initially assessed the absence of frontal sinuses and the presence of unilateral sinuses. In regard to bilateral sinus absence, we found that 3.7% of the sample lacked a frontal sinus, which is similar the estimated global rate of approximately 5% of the population lacking a frontal sinus [27]. This indicates that the sample used in this study is likely representative of an ancestrally global sample of individuals from largely tropic, subtropic, and temperate areas as opposed to studies that focus on cold-adapted arctic populations, which may lack a frontal sinus 25% of the time [3,27].

Concerning unilateral frontal sinus presentations (12.0%), this study found that sinuses were more common on the left side of the cranium and were not impacted by an individual’s assigned sex or population affinity. Previous studies have also noted no differences in the side presentations between assigned sexes [79,80]. While some studies have postulated that population affinity may be responsible for these results, the current study did not find that population affinity impacted the side presentation [27,81,82]. When comparing the side presented in the literature, there seems to be no consensus on which side presents most commonly, globally, or amongst different study populations [19,79,80,82]. The authors of previous studies have postulated similar hypotheses for why the frontal sinus would trend unilaterally as they have in the past to describe overall variation; climate or ancestral adaptive trends [19,79,80,82]. Since there is no obvious patterning in unilateral presentation across studies, this characteristic seems to be more representative of idiosyncratic human variation.

### 4.2. Shape Variation

The EFA results of this study, indicating no statistical significance between morphological clustering and the tested variables, demonstrate that frontal sinus morphology is highly unique and not influenced by assigned sex or population affinity in the study sample. This affirms the results of Goyal et al. [81], who found no significant differences between assigned sexes when assessing morphology, including the number of scallops, the number of partial septa, and whether the frontal sinus was unilateral or bilateral. The idiosyncratic nature of the frontal sinus is further supported by studies that have shown that coded or metric systems for identification purposes are not as reliable as superimposition or side-by-side comparative methods, as the intricacies of frontal sinus morphology have yet to be successfully quantified, categorized, or coded [13,19,28,30,34,46].

Morphological traits typically assessed in the frontal sinus include which side the septum falls, the number of partial septa, scalloping of the arcade, symmetry vs. asymmetry, differences in size, area over the eye orbit, and superiority of the upper border [13,19,23,30,46]. Using EFA and SHAPE v1.3 software, this study was able to quantify these intricacies and thus categorize the general shapes of the frontal sinus into three broad cluster classifications. Results indicated that none of the variables studied affected the overall shape, similar to the findings of Besana & Rogers [23]. Unlike Suman et al. [46], this study did not find that overall shape, including asymmetry, revealed any pattern amongst U.S.-based population affinity groups. Previous studies have determined that coding systems between 14 and 28-digit codes have the capability to aid in identification through exclusion, although they lack the accuracy to positively identify due to the wide variety of traits that individuals could present in combination [13,19,23,28,30,46].

The morphological results of the present study further support the highly idiosyncratic morphology of frontal sinuses and its important role in positive identification as found by Christensen [2,22,29]. Christensen’s [22,29] work with over 300 individuals and 500+ radiographic images used EFA coefficients as quantifiable shape descriptors for the outline of each frontal sinus, arbitrarily terminated just superior to the orbits. Studies previous to Christensen’s had limited sample sizes (e.g., *n* = 32 in Harris et al. [83] and *n* = 35 in Ubelaker [84]), and Christensen [22,29] demonstrated that similarities between replicates in her study were significantly closer in morphology than any similarities between individuals. This confirmed that frontal sinus outlines are unique enough to positively identify individuals using a superimposition method, and practitioners are highly unlikely to incorrectly match antemortem and postmortem radiographs [22]. Christensen [2] additionally demonstrated that the frontal sinus superimposition method is not only reliable enough to meet *Daubert* Standards in court, but that practitioners could provide correct identifications 96% of the time [2]. Thus, the frontal sinus’s unique morphology is unlikely to be caused by sexual dimorphism or population affinity.

### 4.3. Dimensional Variation

It is clear that neither population affinity nor assigned sex alone impacts the dimensions of the frontal sinus, as none of the Pearson’s chi-square tests were statistically significant. More interestingly, when assigned sex and population affinity were tested against the maximum height, maximum depth, and H × W × D, the ANOVA results were statistically significant. Tukey post hoc tests confirmed that assigned sex as a factor and assigned sex in conjunction with population affinity as factors had the most influence on these dimensions. Most of the differences are between AFABs and AMABs of different population affinities (e.g., African-derived AFABs vs. Asian-derived AMABs), and no differences were found between AFABs or AMABS across the population affinities (i.e., all AFABs were similar and all AMABs were similar). The maximum depth demonstrated the most differences in population affinity/assigned sex comparisons, followed by H × W × D, and height, while the maximum width did not show statistically significant population affinity or assigned sex differences. Generally, AMABs tended to have larger frontal sinus depths in comparison to AFABs from different population affinities; however, only the African-derived and Latin American-derived groups were sexually dimorphic in frontal sinus depths. Further, African-derived AFABs were different from all AMAB groups in maximum depth, while Latin American-derived AFABs were different from Latin American-, African-, and Asian-derived AMABs.

This study affirms some previous research findings that the frontal sinus develops differently in individuals from different population affinity groups and assigned sexes. Hacl et al. [36] conducted a dimensional analysis and noted no statistically significant variations between assigned sexes when assessing the height and width of the frontal sinus through their 3D assessment of the frontal sinuses of 22 females and 14 males. The results of the current study also found that frontal sinus width is not sexually dimorphic and height is only dimorphic between different population affinities. Buyuk et al. [33] found that males tended to have larger frontal sinuses overall in their Turkish sample (*n* = 148); however, the authors noted that other similar studies resulted in different findings, and they proposed that age, population, or environmental factors could result in this discrepancy. Verma et al. [26] also found similar results in their South Indian sample of 50 males and 50 females, indicating that males typically had larger frontal sinus heights and widths than females. Both studies approximated the parameters of the frontal sinus by taking multiple measurements of the sinuses and cranium. Buyuk et al. [33] measured the maximum height and width of the frontal sinus as well as the maxillary width, nasal width, cranial width, and antegonial width. Verma et al. [26] measured the maximum right and left frontal sinus heights, maximum right and left widths, and the left, right, and total area of the frontal sinus with an arbitrary inferior border delineated at the top of the eye orbits, similar to Christensen [29] and proposed by Libersa and Faber [26,29,33,85]. Likewise, Hamed et al. [35] used CT scans of 50 females and 50 males between 20–70 years for their assessment of the reliability of the frontal sinus in estimating sex. They found that the best measurement to assess for sexual dimorphism was the right anteroposterior length (i.e., depth), which estimated assigned sex with 67% accuracy, and females presented with smaller measurements than males. Ultimately, these studies were performed on AFABs and AMABs who broadly share the same population histories, which differ somewhat from the present study’s findings that most of the differences are between AFABs and AMABs of different population affinities.

The sexually dimorphic nature of the frontal sinus depth documented in the present study may be related to glabellar morphology. The robusticity of glabella has been used by forensic anthropologists to estimate sex of an individual, with a more pronounced glabella suggesting AMAB [86,87]. This trait may be prominent on the cranium and can typically be seen through the soft tissues of the forehead; thus, clinicians performing gender-affirming facial feminization surgeries focus on the frontal near the frontal sinus [44]. Lee et al. [44] found that the midline of the frontal presents the largest degree of sexual dimorphism, and this decreases laterally. Considering the well-documented sexual dimorphism of glabella externally [75,86,87,88] and its proximity to the frontal sinus, it would be interesting for future research to study whether frontal sinus depths are correlated with nonmetric glabellar trait scores.

### 4.4. Interactive Effects of Sexual Dimorphism and Ancestral Adaptions

The population affinities included in this research were African-, Asian-, European-, and Latin-American derived groups that may reflect residual ancestral adaptations to various tropic, subtropic, and temperate environments. In theory, the colder adapted groups—Asian- and European-derived groups—may have larger overall frontal sinus dimensions than those derived from Africa or Latin America as an adaptation to extreme cold includes pneumatization [89]. However, neither morphological nor dimensional variation differed significantly when looking solely at differences between the population affinities. While African-derived AFABs had the smallest frontal sinus depths compared to AFABs and AMABs from the other population affinities, only comparisons to AMABs were statistically significant. Additionally, when assessing frontal sinus aplasia across populations originating from diverse environments, Aydinliogu et al. [27] found that indigenous Alaskans had the highest rate of absence (25% in males), while Yoshino et al. [19] reported that Japanese males had a rate of absence of 4.8%. Yet, both locations experience some overlapping levels of cold. Additionally, studies have considered paranasal and maxillary sinus volumes as a proxy for climate adaptation, especially when examining Neanderthal crania [37,43]. Noback et al. [37] compared Neanderthal crania to samples from Nubia and Greenland and found that Neanderthal sinuses were closer to the Nubian sample and that volume was not an appropriate indicator of cold climate adaptation. Holton et al. [43] examined broader groups with European, African, and Neanderthal samples and similarly found that volume was not an appropriate proxy when assessing climate adaptation. The current study did not find any statistically significant variation across population affinities in frontal sinus absence and, instead, found that overall, 3.7% of individuals lacked a frontal sinus. Thus, it appears that frontal sinus variation overlaps considerably and does not parse out along population affinity or ancestral lines for individuals living in the U.S. This may be due to overlapping climatic conditions experienced by ancestral groups living in Africa, Asia, Europe, and Latin America—where extreme cold is not represented—and/or to a shared, largely temperate North American environment among descended groups.

The results of this study indicate that sexual dimorphism or ancestral adaptations alone are not correlated with frontal sinus morphological or dimensional variation in this study sample. Instead, the interactive effects of these variables impact the formation and expansion of the frontal sinus, as reflected in the dimensional analyses. AFABs tend to have smaller dimensions when compared to AMABs from different population affinities. However, when comparing AFABs or AMABs within population affinities there is significant overlap, aside frontal sinus depths in African- and Latin American-derived individuals. It is possible that previous authors’ postulations that cranial size or climate adaptations could indeed impact frontal sinus variation when looking at globally extreme groups, but this does not necessarily hold true for groups living in the U.S. The relationship between frontal sinus expansion and correlated environmental temperature is nuanced; the volume of negative space within the frontal might help with thermoregulation and simultaneously be limited by sexually dimorphic cranial sizes. Thus, it seems that if a group from a cold climate benefits from a larger frontal sinus, the frontal sinus will expand but only as far as it can without compromising the integrity of the frontal. Likewise, there is no need for a frontal sinus to form as expansively in a hotter climate in smaller individuals and it might even be detrimental to their homeostasis. Indeed, African-derived AFABs (e.g., those descended from biogeographically warm locations) had the smallest frontal sinus depths compared to all other groups; however, this does not hold true for African-derived AMABs. Following Bergmann’s and Allen’s rules, it would be interesting to test if there is a correlation between overall axial surface area or appendage length of AFAB vs. AMAB individuals and frontal sinus dimensions to determine if latitudinal variation and climate adaptation really is the driving factor [90,91,92,93,94]. Globally, AFABs tend to be shorter and smaller than AMABs, so it would be noteworthy if there were internal compensatory thermoregulation mechanisms. It is also possible that these ancestral adaptations are no longer evident in modern humans as populations are more geographically mobile and climates have shifted and changed in severity. Thus, for groups living in the U.S., sexual dimorphism appears to be more impactful in frontal sinus dimensional variation than ancestral adaptations.

### 4.5. The Use of CT Scans and Image Orientation/Quality

The analysis of CT images represents a valuable method for ascertaining potential environmental or biological impacts on frontal sinus formation. CT scans allow for nuanced morphological and metric analyses to study variation and are increasingly common in medical records. All files received for this study had both clinically standardized coronal slices and sagittal/parasagittal slices of the cranium allowing for complete analysis. Besides noting the outermost border of the frontal sinus, having an abundance of images from one individual allows for more clarity as to how the frontal sinus is oriented within the cranium and how it relates into other structures. This eliminated the necessity of using an arbitrary lower boundary of the frontal sinus at the top of the eye orbits in the morphological analysis as done in Christensen’s [2,22,29] radiographic analyses of frontal sinus morphology. The arbitrary orbital cutoff is necessary with radiographs due to the obfuscation of the inferior sinus by a confluence of bony structures. By progressively outlining each 1.5 mm CT slice, this study was able to delineate the inferior border of the frontal sinus, allowing for an easier and more complete visualization of where exactly the frontal sinus plateaus and transforms into these other structures.

**Figure 7:**
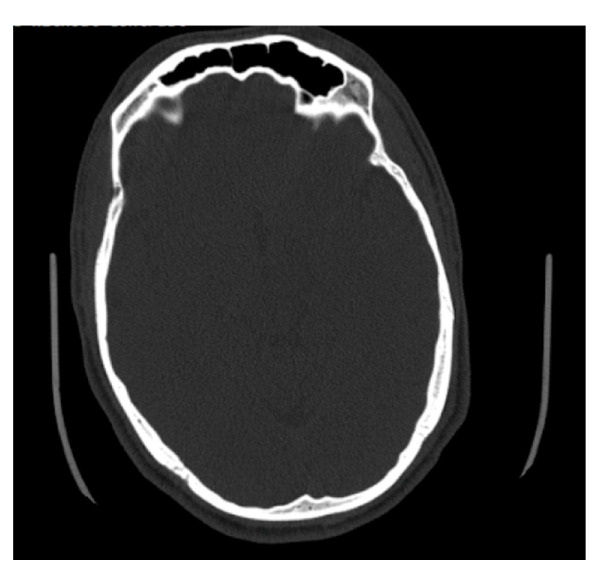
Example of an axial CT image slice useful for measuring frontal sinus depths.

Some of the files received from BMC included slices on the axial plane in addition to the coronal and sagittal orientations (Figure 7). The methods used to determine maximum depth in this study were completed on the sagittal slices; however, the axial slices were significantly better suited for efficiently determining the maximal depth, and in future studies, the axial plane is recommended to measure maximum depth. The axial plane limits any discrepancies in determining where the frontal sinus connects with the nasal cavity and ethmoid sinus, and instead presents a clearer representation of the most anterior and posterior portions of the frontal bone. This orientation also allows for both left and right portions of the frontal sinus to be viewed simultaneously, allowing for an easier estimation of maximum depth. Simple procedures and standardized methods are critical to this kind of analysis. On radiographs, the analyst does not have to estimate the posterior border of the frontal sinus or demarcate where the frontal sinus transforms into other anatomical structures. While this skill is not difficult to develop, it is a skill that needs to be practiced. The axial orientation would eliminate this concern when taking a maximum depth of the frontal sinus, but the concern is still present when deciding which coronal slices to use for tracing.

The methods used in this study allowed for a 2D analysis of the frontal sinus anterior-posterior perimeter captured from CT scans, which typically give three dimensions of information about anatomical structures. This study adds to the body of knowledge regarding the frontal sinus outline on CT scans using clinical data. It should be noted that this methodology was time consuming, as all layers of the CT scans that had visible portions of the frontal sinus were traced to collect an accurate representation of the outermost perimeter of the frontal sinus for each individual. As an explorative method, tracing and quantifying CT images has illuminated some intricacies of frontal sinus variation that traditional radiographs are unable to capture. In particular, this method captured the entirety of the frontal sinus, rather than focusing only on the area superior to the orbital margins. It would be interesting for future research to determine if superimposition or side-by-side comparative methods are accurate for identification between frontal sinuses captured on antemortem CT scans and postmortem radiographs.

### 4.6. Limitations of Study

The current study analyzed images from individuals with varied ancestral backgrounds in order to examine the possible relationship between residual ancestral climate adaptations and frontal sinus morphology and dimensions. One issue is that self-reported ancestral backgrounds in clinical data collection are quite broad, only allowing for large, generalized groups. The ancestral biogeographic origins for the groups analyzed in the present study—that is, Africa, Asia, Europe, and Latin America—overlap to varying extents in temperate, subtropic, and tropic climates. Moreover, the individuals included in the present study all share a uniquely North American climate and environment. In order to further explore relationships between climate adaptations and frontal sinus variation, future studies should aim to collect sample images from groups that do not overlap in climate. Additionally, it would be interesting to study if the climate where ancestral adaptations could occur is most important or if the climate that an individual develops in is the causational factor. The frontal sinus develops prenatally through 20 years of age; thus, it is possible that the idiosyncrasies develop in response to the environment in combination with genetics instead of a purely genetic pathway. This study assessed individuals currently residing in Massachusetts, and future studies following similar methods on global populations would help to explore how an individual’s environment during growth and development impacts frontal sinus variation.

This study did not examine intra- or inter-observer error for the frontal sinus outline tracings or for the dimensional data due, in part, to the primary goal of exploring sinus variation rather than developing discriminatory methods to predict assigned sex or population affinity in forensic casework. Additionally, the time-consuming nature of data collection for EFA precluded a subsequent reanalysis. Thus, future EFA analyses on the frontal sinus would benefit from intra- and inter-observer error studies. However, previous work has demonstrated that measurements taken on CT scans are reliable [75,95]. For example, Kelley and Tallman [75] found that standard cranial measurements from CT scans produced intraclass correlation coefficients (ICC) of 0.777 to 0.989 and that the lowest ICC values were measurements that included suture landmarks, which can be obliterated and difficult to locate.

This study used clinically derived cranial CT scans that were not necessarily oriented on the anthropologically preferred Frankfurt Horizonal Plane [22,47]. However, clearly rotated skulls that would produce distorted outlines and measurements were removed prior to analysis. While Butaric et al. [47] found that 5% deviations in CT skull orientation did not dramatically impact subsequent sinus outlines, measurements, or positive matches, minor inter-individual deviations in head position may have minimally impacted the results presented here. Additionally, a very slight error may have been introduced during the manually digitized tracing of the CT image slices. To mitigate errors introduced by manually outlining sinuses, Butaric et al. [47] used 3D models obtained from 3DSlicer.

Lastly, while the present study was unable to quantify a highly precise volume measurement of the frontal sinus due to the limitations of the DICOM software—and instead used H × W × D—volume calculation now is possible with a new statistical analysis package that was recently created in R by Veneziano [96]. The package, called IndianaBones, allows analysts to compute volume from 3D stacked images. The use of this software may produce interesting data to follow up the current study as it would be possible to determine more accurate volume representations.

## 5. Conclusions

The intricacies of the frontal sinus have been an essential tool used in positive identifications by forensic anthropologists for close to a century, but the cause of the unique development of the sinus remains unknown. This study analyzed both morphological and dimensional variations in a U.S. sample of individuals of differing population affinities and assigned sexes. The EFA results indicated that there was no significant clustering morphologically across assigned sex, population affinities, or both of these variables together. When analyzing the dimensional data, the results indicated that assigned sex as a factor and assigned sex and population affinity together as a factor impacted the maximum depth, H × W × D, and maximum height. However, the majority of differences were found among AFABs and AMABs from different population affinities and no differences were observed between AFABS or between AMABs from different population affinities. Additionally, the depth of the sinus is the most sexually dimorphic dimension, and width exhibited no differences between AFABs and AMABs. This study also found that 3.7% of the sample lacked a frontal sinus and that 12.0% in the dimensional analysis had as unilateral frontal sinuses with the vast majority presenting on the left side of the cranium.

While this study affirms the idiosyncratic nature of the frontal sinus and its use in forensic anthropology, further research is warranted to address the question of why it forms uniquely to an individual. In the U.S., sinus variation does not fall along population affinity lines, suggesting that there was significant overlap in ancestral temperate, subtropic, and tropic climates, or alternately, that ancestral climate adaptations no longer impact variation, particularly in modern individuals from a shared U.S. environment. Moreover, sexual dimorphism appears to play a significant role in driving frontal sinus depth and height variation between broad population affinity groups. In particular, the interplay of sexual differences and ancestral climate adaptations has a significant impact on the dimensions of the frontal sinus; however, a clear pattern has yet to be illuminated.

## Figures and Tables

**Table 1:** Sample sizes used in dimensional and morphological analyses.

Initial Sample *n* = 325	
AFAB *n* = 176 African derived *n* = 45 Asian derived *n* = 44 European derived *n* = 45 Latin American derived *n* = 42	AMAB *n* = 149 African derived *n* = 27 Asian derived *n* = 31 European derived *n* = 48 Latin American derived *n* = 43
Frontal sinuses absent *n* = 12 ↓	
AFAB *n* = 6 African derived *n* = 3 Asian derived *n* = 0 European derived *n* = 1 Latin American derived *n* = 2	AMAB *n* = 6 African derived *n* = 2 Asian derived *n* = 0 European derived *n* = 1 Latin American derived *n* = 3
Sample used in dimensional analyses *n* = 313	
AFAB *n* = 170 African derived *n* = 42 Asian derived *n* = 44 European derived *n* = 44 Latin American derived *n* = 40	AMAB *n* = 143 African derived *n* = 25 Asian derived *n* = 31 European derived *n* = 47 Latin American derived *n* = 40
Frontal sinuses not connected medially *n* = 6 ↓	
AFAB *n* = 3 African derived *n* = 1 Asian derived *n* = 1 European derived *n* = 0 Latin American derived *n* = 1	AMAB *n* = 3 African derived *n*= 3 Asian derived *n* = 0 European derived *n* = 0 Latin American derived *n* = 0
Sample used in morphological analyses *n* = 307	
AFAB *n* = 167 African derived *n* = 41 Asian derived *n* = 43 European derived *n* = 44 Latin American derived *n* = 39	AMAB *n* = 140 African derived *n* = 22 Asian derived *n* = 31 European derived *n* = 47 Latin American derived *n* = 40

**Table 2:** Pearson’s chi-square tests of assigned sex and population affinity vs. the dimensional variables (maximum height, maximum width, maximum depth, and H × W × D).

Variables	Results	Statistical Significance
Assigned sex vs. maximum height	X^2^ = 290.88 df = 291 *p*-value = 0.4909	Fail to reject null hypothesis
Assigned sex vs. maximum width	X^2^ = 310.99 df = 306 *p*-value = 0.4100	Fail to reject null hypothesis
Assigned sex vs. maximum depth	X^2^ = 273.45 df = 274 *p*-value = 0.4980	Fail to reject null hypothesis
Population affinity vs. maximum height	X^2^ = 879.17 df = 873 *p*-value = 0.4351	Fail to reject null hypothesis
Population affinity vs. maximum width	X^2^ = 914.2 df = 918 *p*-value = 0.5292	Fail to reject null hypothesis
Population affinity vs. maximum depth	X^2^ = 850.28 df = 822 *p*-value = 0.2401	Fail to reject null hypothesis
Assigned sex vs. H × W × D	X^2^ = 313 df = 312 *p*-value = 0.4734	Fail to reject null hypothesis
Population affinity vs. H × W × D	X^2^ = 939 df = 936 *p*-value = 0.4663	Fail to reject null hypothesis

**Table 3:** ANOVA tests of assigned sex and population affinity vs. the dimensional variables (maximum height, maximum width, maximum depth, and H × W × D).

Variables	Results	Statistical Significance
Assigned sex and population affinity vs. maximum height	Residual Deviance = 9.499 df = 3 *p*-value = −1.0658 × 10^−8^	Reject null hypothesis
Assigned sex and population affinity vs. maximum width	Residual Deviance = 418.7 df = 3 *p*-value = 0.223	Fail to reject hypothesis
Assigned sex and population affinity vs. maximum depth	Residual Deviance = 9.499 df = 3 *p*-value = −1.0658 × 10^−8^	Reject null hypothesis
Assigned sex and population affinity vs. H × W × D	Residual Deviance = 9.499 df = 3 *p*-value = −1.0658 × 10^−8^	Reject null hypothesis

**Table 4:** Tukey post hoc tests of the statistically significant ANOVA tests.

Variables	Results	Statistical Significance	Significant Adjusted *p*-Values
Assigned sex and population affinity vs. maximum height	Assigned sex as a factor *p*-value = 0.00157	Reject null hypothesis	AFAB vs. AMAB: *p*-value = 0.0015679
	Population affinity as a factor *p*-value = 0.13536	Fail to reject null hypothesis	-
	Assigned sex and population affinities as factors *p*-value = 0.01191	Reject null hypothesis	African-derived AFAB vs. Latin-derived AMAB: *p*-value = 0.0296342; Asian-derived AFAB vs. African-derived AMAB: *p*-value = 0.0204191; Asian-derived AFAB vs. Latin-derived AMAB: *p*-value = 0.0008630
Assigned sex and population affinity vs. maximum depth	Assigned sex as a factor *p*-value = 4.3 × 10^−10^	Reject null hypothesis	AFAB vs. AMAB: *p*-value = 4.3 × 10^−10^
	Population affinity as a factor *p*-value = 0.499	Fail to reject null hypothesis	-
	Assigned sex and population affinities as factors *p*-value = 0.146	Reject null hypothesis	African-derived AFAB vs. African-derived AMAB: *p*-value = 0.0059593; African-derived AFAB vs. Asian-derived AMAB: *p*-value = 0.0022128; African-derived AFAB vs. Latin-derived AMAB: *p*-value = 0.0000058; African-derived AFAB vs. European-derived AMAB: *p*-value = 0.0151888; Latin-derived AFAB vs. African-derived AMAB: *p*-value = 0.0303812; Asian-derived AFAB vs. Latin-derived AMAB: *p*-value = 0.0057280; Latin-derived AFAB vs. Asian-derived AMAB: *p*-value = 0.0135587; Latin-derived AFAB vs. Latin-derived AMAB: *p*-value = 0.0000928; European-derived AFAB vs. Latin-derived AMAB: *p*-value = 0.0055286
Assigned sex and population affinity vs. (H × W × D)	Assigned sex as a factor *p*-value = 3.25 × 10^−5^	Reject null hypothesis	AFAB vs. AMAB: *p*-value = 0.0000325
	Population affinity as a factor *p*-value = 0.8747	Fail to reject null hypothesis	-
	Assigned sex and population affinities as factors *p*-value = 0.0177	Reject null hypothesis	African-derived AFAB vs. African-derived AMAB: *p*-value = 0.0033445; African-derived AFAB vs. Asian-derived AMAB: *p*-value = 0.0200277; African-derived AFAB vs. Latin-derived AMAB: *p*-value = 0.0117864; Asian-derived AFAB vs. African-derived AMAB: *p*-value = 0.0336140

**Table 5:** Descriptive statistics for the frontal sinus maximum height by population affinity and assigned sex.

Maximum Height (in mm)				
Group	*n*	Mean	Range	Standard Deviation
African-derived AFABs	42	22.98 ^a^	10.53–38.66	7.36
African-derived AMABs	27	28.44 ^b^	8.68–58.71	11.84
African-derived AFABs and AMABs	69	25.12	8.68–58.71	9.67
Asian-derived AFABs	43	21.13 ^b, c^	7.02–43.06	9.83
Asian-derived AMABs	29	27.53	11.53–58.81	10.28
Asian-derived AFABS and AMABs	72	23.71	7.02–58.81	10.43
European-derived AFABs	43	26.75	9.45–44.43	8.05
European-derived AMABs	46	24.68	12.58–41.48	7.05
European-derived AFABs and AMABs	89	25.68	9.45–44.43	7.58
Latin American-derived AFABs	40	25.52	9.31–44.83	8.09
Latin American-derived AMABs	43	29.21 ^a, c^	13.36–50.71	9.41
Latin American-derived AFABs and AMABs	83	27.43	9.31–50.71	8.94
All AFABs	168	24.08 ^d^	7.02–44.83	8.61
All AMABs	145	27.29 ^d^	8.68–58.81	9.53

^a^ Statistically significantly different (*p*-value = 0.0296342); ^b^ Statistically significantly different (*p*-value = 0.0204191); ^c^ Statistically significantly different (*p*-value = 0.0008630); ^d^ Statistically significantly different (*p*-value = 0.0015679) (see Table 4).

**Table 6:** Descriptive statistics for the frontal sinus maximum width by population affinity and assigned sex.

Maximum Width (in mm)				
Group	*n*	Mean	Range	Standard Deviation
African-derived AFABs	42	50.08	14.89–79.39	15.99
African-derived AMABs	27	57.38	10.62–108.12	26.66
African-derived AFABs and AMABs	69	52.94	10.62–108.12	20.95
Asian-derived AFABs	43	49.89	9.95–98.15	23.07
Asian-derived AMABs	29	58.06	13.15–96.01	22.55
Asian-derived AFABs and AMABs	72	53.18	9.95–98.15	23.06
European-derived AFABs	43	58.53	20.67–114.02	21.48
European-derived AMABs	46	55.07	15.46–95.01	19.09
European-derived AFABs and AMABs	89	56.74	15.46–114.02	20.24
Latin American-derived AFABs	40	56.19	26.48–87.64	18.12
Latin American-derived AMABs	43	56.29	20.06–88.45	17.97
Latin American-derived AFABs and AMABs	83	56.24	20.06–88.45	17.93
All AFABs	168	53.65	9.95–114.02	20.10
All AMABs	145	56.46	10.62–108.12	20.89

**Table 7:** Descriptive statistics for the frontal sinus maximum depth by population affinity and assigned sex.

Maximum Depth (in mm)				
Group	*n*	Mean	Range	Standard Deviation
African-derived AFABs	42	9.85 ^a, b, c, d^	5.69–15.04	2.49
African-derived AMABs	27	13.27 ^a, e^	5.53–25.61	5.28
African-derived AFABs and AMABs	69	11.19	5.53–25.61	4.15
Asian-derived AFABs	43	11.16 ^f^	3.65–23.19	4.98
Asian-derived AMABs	29	13.44 ^b, g^	8.29–21.78	4.01
Asian-derived AFABs and AMABs	72	12.08	3.65–23.19	4.72
European-derived AFABs	43	11.15 ^i^	4.36–18.04	3.15
European-derived AMABs	46	12.59 ^d^	8.78–22.47	3.10
European-derived AFABs and AMABs	89	11.90	4.36–22.47	3.19
Latin American-derived AFABs	40	10.27 ^e, g, h^	6.36–15.63	2.26
Latin American-derived AMABs	43	14.15 ^c, f, h, i^	7.02–28.26	4.18
Latin American-derived AFABs and AMABs	83	12.28	6.36–49.84	3.90
All AFABs	168	10.62 ^j^	3.65–23.19	3.43
All AMABs	145	13.35 ^j^	5.53–28.26	4.08

^a^ Statistically significantly different (*p*-value = 0.0059593); ^b^ Statistically significantly different (*p*-value = 0.0022128); ^c^ Statistically significantly different (*p*-value = 0.0000058); ^d^ Statistically significantly different (*p*-value = 0.0151888); ^e^ Statistically significantly different (*p*-value = 0.0303812); ^f^ Statistically significantly different (*p*-value = 0.0057280); ^g^ Statistically significantly different (*p*-value = 0.0135587); ^h^ Statistically significantly different (*p*-value = 0.0000928); ^i^ Statistically significantly different (*p*-value = 0.0055286); ^j^ Statistically significantly different (*p*-value = 4.3 × 10^−10^) (see Table 4).

## Data Availability

The data for this study are kept by the first author.

## Supplementary Materials

The following supporting information can be downloaded at: https://www.mdpi.com/article/10.3390/biology11081145/s1, Video S1: CT slices with tracing of frontal sinus outlines. Video S2: Composite of traced layers of the frontal sinus.
